# Supplementary material for: Systemic immune dysregulation in hypertensive disorders of pregnancy persists years after delivery
Source: Front Immunol. 2026 Feb 5;17:1716809. doi: 10.3389/fimmu.2026.1716809 (PMC12916653; doi:10.3389/fimmu.2026.1716809)
Supplement: Supplementary file 4 [file Table1.docx]

**Table S1 – Impact on model performance when incorporating prior immunological knowledge**

AUROC = Area Under the Receiver Operating Characteristics curve. AUPRC = Area Under the Precision-Recall Curve

|  | AUROC | AUPRC | p-value |
| --- | --- | --- | --- |
| Antepartum Model | | | |
| Model Without Penalization | 0.8 | 0.789 | 0.000036 |
| Best Penalized Model | 0.814 | 0.795 | 0.00002 |
| Postpartum Model | | | |
| Model Without Penalization | 0.743 | 0.72 | 0.00021 |
| Best Penalized Model | 0.757 | 0.729 | 0.00009 |
| Midlife Model | | | |
| Model Without Penalization | 0.674 | 0.664 | 0.00032 |
| Best Penalized Model | 0.692 | 0.666 | 0.00007 |
