# Supplementary material for: Systemic immune dysregulation in hypertensive disorders of pregnancy persists years after delivery
Source: Front Immunol. 2026 Feb 5;17:1716809. doi: 10.3389/fimmu.2026.1716809 (PMC12916653; doi:10.3389/fimmu.2026.1716809)
Supplement: Supplementary file 5 [file Table2.docx]

**Table S2 – Confounder analysis**

|  | coefficient | Standard error | $t$ value | $Pr>\vert t\vert$ | Significance |
| --- | --- | --- | --- | --- | --- |
| Antepartum Model | | | | | |
| age | -0.0117277 | 0.00819156 | -1.4316793 | 0.1576026 | NS |
| BMI at study visit | 0.01127746 | 0.00857463 | 1.31521236 | 0.19361458 | NS |
| parity | 0.04357068 | 0.05352212 | 0.81406858 | 0.41893542 | NS |
| study visit timing (GA) | -0.0288742 | 0.01020742 | -2.82875 | 0.00640655 | ** |
| GDM | 0.16399123 | 0.1680492 | 0.97585252 | 0.33319024 | NS |
| systolic | 0.00705574 | 0.00278467 | 2.53377856 | 0.01400802 | * |
| diastolic | 0.0064868 | 0.00568303 | 1.14143336 | 0.25838046 | NS |
| modelPreds | 0.33157814 | 0.13035584 | 2.54363859 | 0.01365808 | * |
| Postpartum Model | | | | | |
| age | -0.0100543 | 0.01322547 | -0.7602203 | 0.4497128 | NS |
| BMI at study visit | 0.00789642 | 0.00976408 | 0.80872132 | 0.42145522 | NS |
| parity | 0.03382038 | 0.06688209 | 0.50567167 | 0.61469881 | NS |
| study visit timing (weeksSinceDelivery) | 0.01221979 | 0.00520066 | 2.3496608 | 0.02165802 | * |
| GDM | 0.41748391 | 0.19546686 | 2.13582956 | 0.03624407 | * |
| systolic | 0.00020336 | 0.0057866 | 0.03514355 | 0.97206674 | NS |
| diastolic | 0.0023361 | 0.00928791 | 0.25152017 | 0.80215957 | NS |
| modelPreds | 0.44871433 | 0.19805219 | 2.26563676 | 0.02661499 | * |
| Midlife Model | | | | | |
| age | -0.0228414 | 0.00756955 | -3.0175404 | 0.00304682 | ** |
| BMI at study visit | 0.00400979 | 0.00839975 | 0.47737095 | 0.63386994 | NS |
| parity | 0.01487917 | 0.03749529 | 0.39682755 | 0.69212125 | NS |
| study visit timing (yearsSinceDelivery) | 0.01491857 | 0.01294151 | 1.15276857 | 0.25104248 | NS |
| GDM | 0.57227264 | 0.19833578 | 2.88537266 | 0.00455278 | ** |
| systolic | 0.00339593 | 0.00472445 | 0.71879884 | 0.47350717 | NS |
| diastolic | 0.00787966 | 0.00610156 | 1.29141571 | 0.19876635 | NS |
| modelPreds | 0.42593933 | 0.16310091 | 2.61150796 | 0.01003427 | * |

Variables examined for potential confounding do not create a major impact on the outcome of the multivariate models. Model predictions remain significant for each cohort when considering potential confounding variables. GA = gestational age; GDM = gestational diabetes mellitus. BMI = body mass index. NS = not significant. Significance codes: * < 0.05, ** < 0.01.
