# Supplementary material for: Systemic immune dysregulation in hypertensive disorders of pregnancy persists years after delivery
Source: Front Immunol. 2026 Feb 5;17:1716809. doi: 10.3389/fimmu.2026.1716809 (PMC12916653; doi:10.3389/fimmu.2026.1716809)
Supplement: Supplementary file 6 [file Table3.docx]

**Table S3** – **Informative model features**. Features selected for each model, along with their coefficients in the final model. Negative coefficients indicate decreased values in cases compared to controls.

| AP Model Feature | Coefficient | AP Model Feature - continued | Coefficient |
| --- | --- | --- | --- |
| ncMCs_VDAC1_IL18 | 2.408 | FceRIanegDC_CXCR4_IL246 | -0.511 |
| Ki67CD8Tcell_HK2_IL18 | 1.676 | gdTcell_pP38_IL18 | -0.572 |
| ncMCs_GLUT1_IL18 | 1.669 | DC_CXCR4_LPS | -0.597 |
| Treg_HK2_IL18 | 1.524 | Th2_pP38_IL18 | -0.603 |
| Ki67CD4Tcell_H3K27me3_IL18 | 1.268 | DC_VDAC1_Unstim | -0.704 |
| Th2_CXCR4_Unstim | 1.005 | FceRIaposDC_CD62L_LPS | -0.73 |
| Eosinophil_pERK1-2_IL18 | 0.982 | Neutrophil_pP38_IL18 | -0.869 |
| DC_pNFkB_Unstim | 0.637 | CD8Tcell_CD62L_IL18 | -1.574 |
| Neutrophil_GLUT1_LPS | 0.599 | DC_CXCR4_IL18 | -5.101 |
| NKTlike_H3K27me3_IL18 | 0.408 | PP Model Feature | Coefficient |
| Ki67CD8Tcell_CD62L_IL18 | 0.339 | NK_CXCR4_IL18 | 0.035 |
| CD4CM_frequency_Unstim | 0.328 | Bcells_CXCR4_IL18 | -0.659 |
| Treg_HK2_IL246 | 0.322 | NKTlike_CXCR4_IL18 | -0.774 |
| CD8CM_pP38_IL18 | 0.303 | Treg_CXCR4_IL18 | -0.835 |
| Neutrophil_CD62L_LPS | 0.302 | ML Model Feature | Coefficient |
| FceRIaposDC_pERK1-2_LPS | 0.235 | Ki67CD8Tcell_pCREB_IL246 | 1.146 |
| gdTcell_H3K27me3_IL18 | 0.227 | Treg_CPT1a_Unstim | 0.88 |
| DC_VDAC1_LPS | 0.206 | Basophil_pP38_LPS | 0.523 |
| Treg_CPT1a_Unstim | 0.095 | Ki67CD8Tcell_pCREB_IL18 | 0.272 |
| Treg_pPLCg1_Unstim | 0.086 | NKTlike_frequency_Unstim | 0.123 |
| intMCs_GLUT1_IL18 | 0.083 | Granulocytes_CD62L_Unstim | -0.214 |
| CD4Trm_pSTAT1_IL246 | -0.026 | Neutrophil_CD62L_Unstim | -0.346 |
| Bcells_PD-L1_IL18 | -0.064 | Granulocytes_pCREB_LPS | -0.415 |
| Ki67CD8Tcell_CD62L_Unstim | -0.221 | NKTlike_pSTAT6_IL246 | -0.442 |
| CD16+_pCREB_LPS | -0.238 | Neutrophil_pCREB_LPS | -0.477 |
| Th1_CD62L_IL246 | -0.303 | Basophil_pSTAT6_IL246 | -0.545 |
| Neutrophil_VDAC1_Unstim | -0.354 | CD16-_H3K27me3_IL246 | -0.639 |
| DC_pSTAT3_IL246 | -0.483 | Ki67CD8Tcell_CXCR4_IL246 | -0.749 |
