# Supplementary material for: Systemic immune dysregulation in hypertensive disorders of pregnancy persists years after delivery
Source: Front Immunol. 2026 Feb 5;17:1716809. doi: 10.3389/fimmu.2026.1716809 (PMC12916653; doi:10.3389/fimmu.2026.1716809)
Supplement: Supplementary file 7 [file Table4.docx]

**Table S4** – **Correlation of persistent immune features with risk factors of cardiovascular disease**. Spearman correlation coefficients. BMI = body mass index; AP = antepartum; PP = postpartum; ML= midlife

|  | | BMI at study visit | Systolic | Diastolic |
| --- | --- | --- | --- | --- |
| Bcells frequency Unstim | AP | -0.0571339 | 0.29058527 | 0.29139088 |
|  | PP | -0.0160175 | 0.14572898 | -0.1083655 |
|  | ML | 0.18071013 | -0.0124464 | 0.03953211 |
| cMCs pSTAT3 IL246 | AP | -0.245465 | -0.2587852 | -0.2482721 |
|  | PP | -0.4198393 | -0.1620847 | -0.1147438 |
|  | ML | -0.4498219 | -0.142363 | -0.1198914 |
