# Supplementary material for: Systemic immune dysregulation in hypertensive disorders of pregnancy persists years after delivery
Source: Front Immunol. 2026 Feb 5;17:1716809. doi: 10.3389/fimmu.2026.1716809 (PMC12916653; doi:10.3389/fimmu.2026.1716809)
Supplement: Supplementary file 9 [file Image2.pdf]

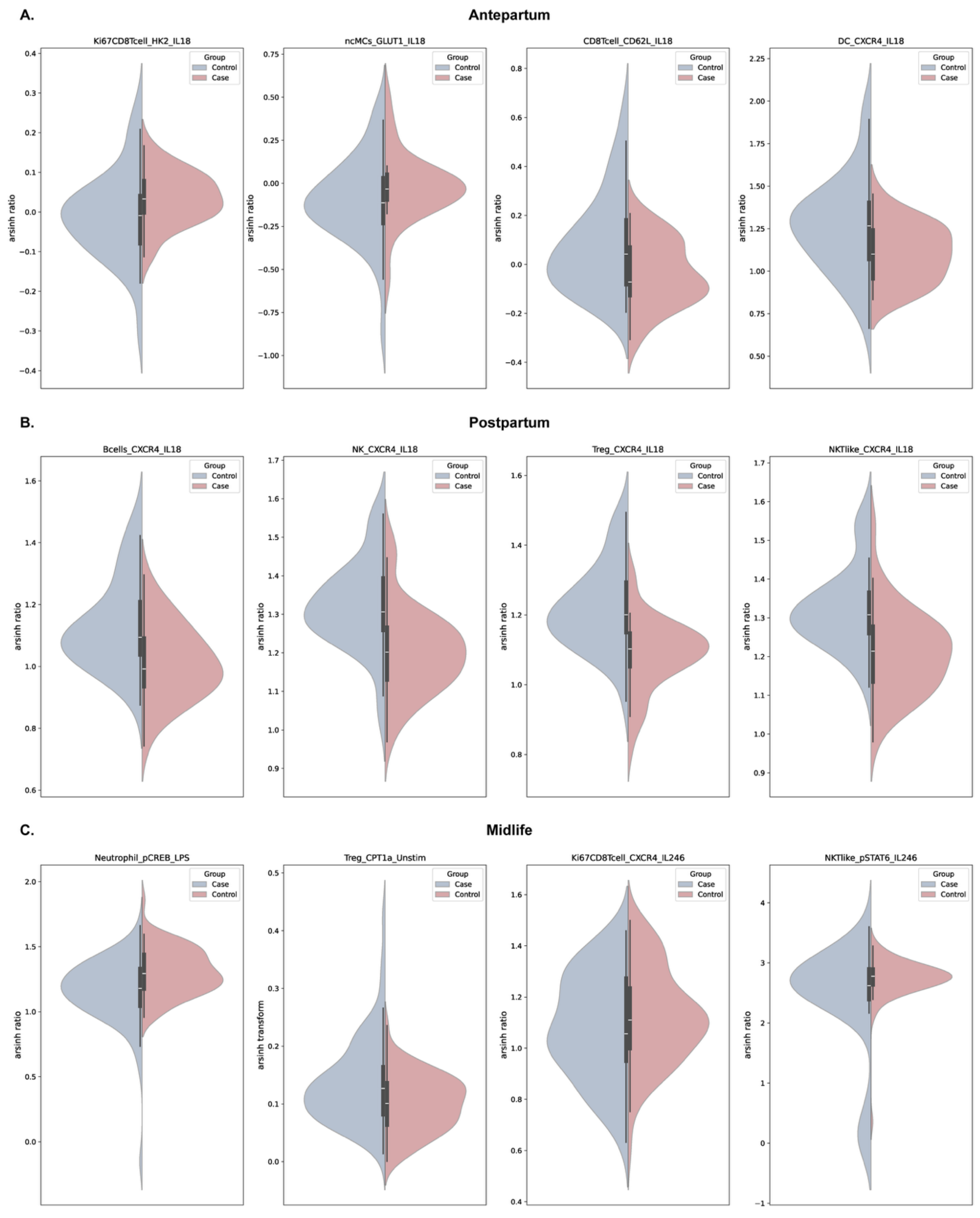

Figure S2 - Informative model features.

Violin plots highlight representative model features for each cohort. Values indicate median marker expression. **A.** Selected features in the antepartum model. **B.** Selected features in the postpartum model. **C.** Selected features in the midlife model. ncMCs = non-classical monocytes; DC = dendritic cell; Treg = regulatory T cell.
