# Supplementary material for: Systemic immune dysregulation in hypertensive disorders of pregnancy persists years after delivery
Source: Front Immunol. 2026 Feb 5;17:1716809. doi: 10.3389/fimmu.2026.1716809 (PMC12916653; doi:10.3389/fimmu.2026.1716809)
Supplement: Supplementary file 10 [file Image3.pdf]

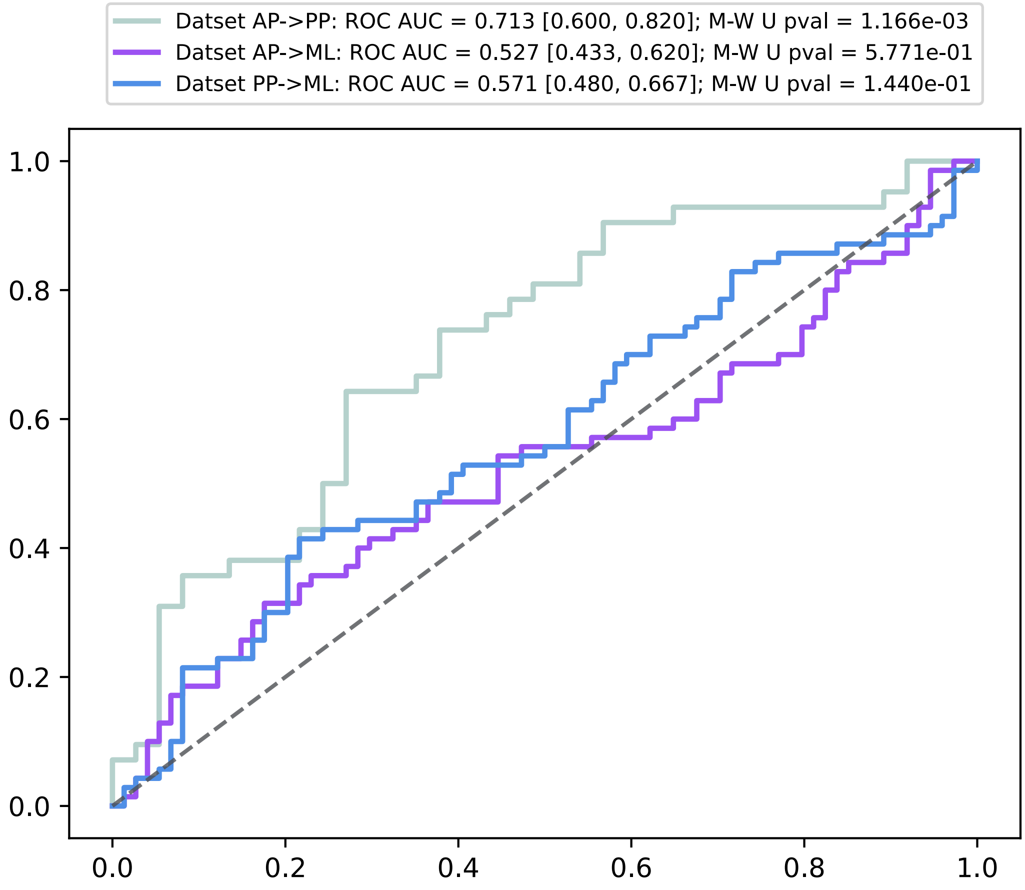

Figure S3 – Model performance when applied to later timepoints
Performance of models when AP or PP models are applied to predict outcomes in later cohorts, displayed as Area Under the Receiver Operator Characteristic curves. AP→PP = AP model used to predict outcomes in PP cohort; AP→ML = AP model used to predict outcomes in ML cohort; PP→ML = PP model used to predict outcomes in ML cohort. AP = antepartum; PP = postpartum; ML= midlife
