# Supplementary material for: Systemic immune dysregulation in hypertensive disorders of pregnancy persists years after delivery
Source: Front Immunol. 2026 Feb 5;17:1716809. doi: 10.3389/fimmu.2026.1716809 (PMC12916653; doi:10.3389/fimmu.2026.1716809)
Supplement: Supplementary file 12 [file Image5.pdf]

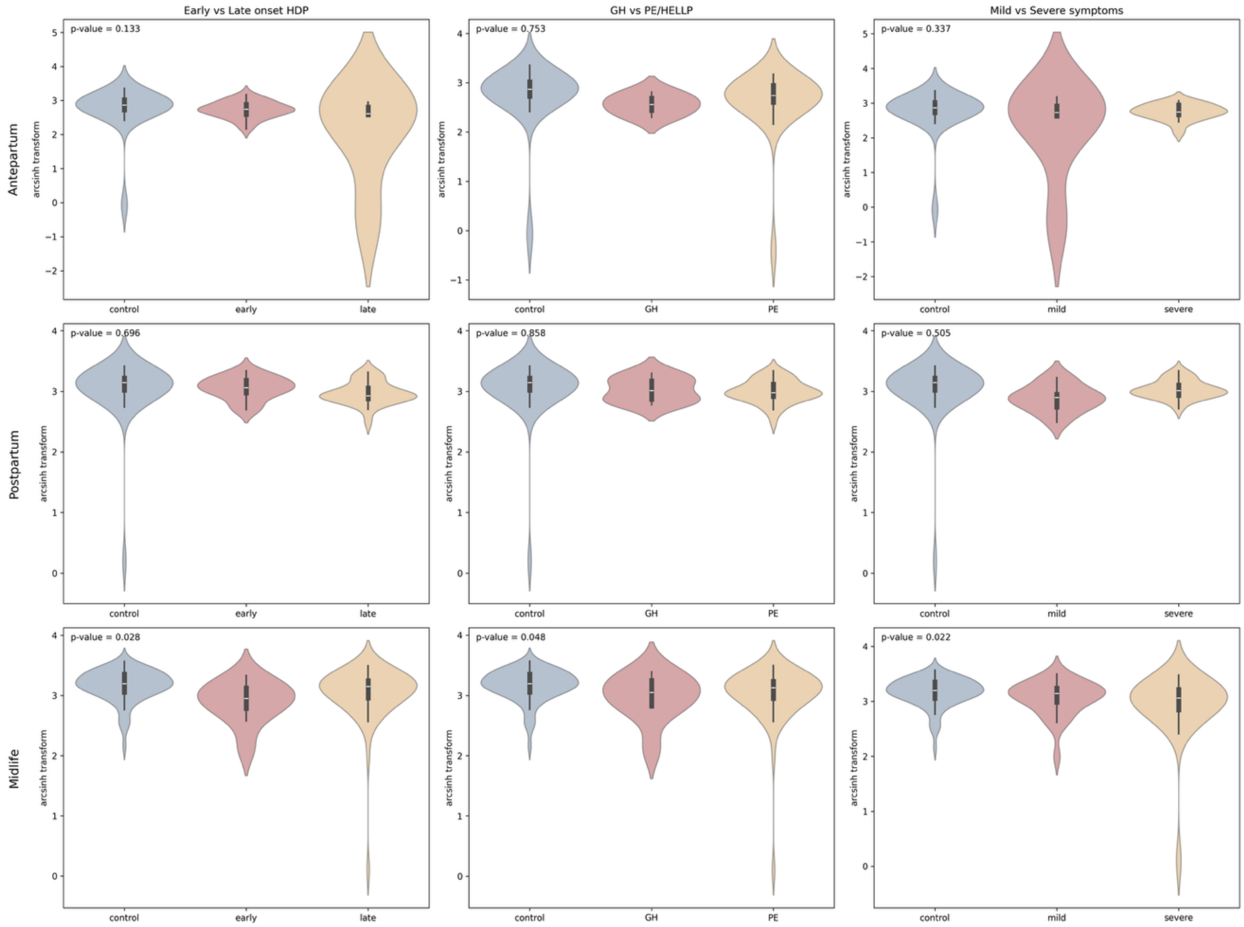

Figure S5 – pSTAT3 expression in cMC after IL-2/IL-4/IL-6 stimulation stratified by HDP subsets

Violin plots representing median pSTAT3 expression in classical monocytes (cMC) after IL-2/IL-4/IL-6 stimulation stratified by subsets of hypertensive disorders of pregnancy (HDP) per timepoint. Subsets include 1) early (≤34 weeks) versus late (>34 weeks) onset of HDP, 2) gestational hypertension (GH) versus preeclampsia (PE) and Hemolysis, Elevated Liver enzymes and Low Platelets (HELLP) syndrome, and 3) mild (GH + PE with mild features) versus severe (PE with severe features + HELLP) HDP symptoms. P-values derived from ANOVA test.
