# Supplementary material for: Systemic immune dysregulation in hypertensive disorders of pregnancy persists years after delivery
Source: Front Immunol. 2026 Feb 5;17:1716809. doi: 10.3389/fimmu.2026.1716809 (PMC12916653; doi:10.3389/fimmu.2026.1716809)
Supplement: Supplementary file 8 [file Image1.pdf]

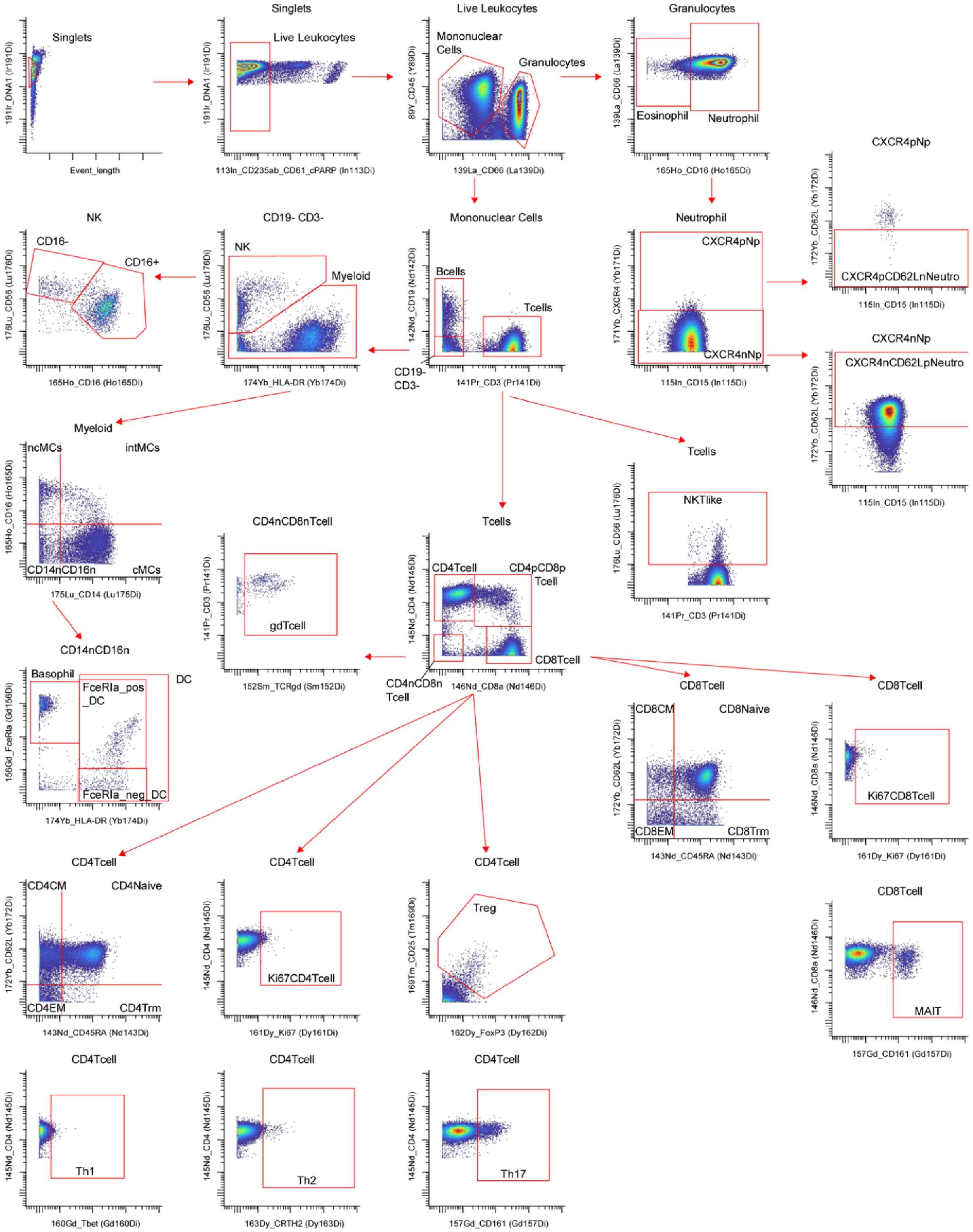

Figure S1 – Immune cell gating strategy. Bivariate dot plots for the identification of immune cell populations. Title above each dot plot represents the input gate. NK = natural killer cell; ncMCs = non-classical monocytes; intMCs = intermediate monocytes; cMCs = classical monocytes; DC = dendritic cell; NKTlike = natural killer like T cell; CD8CM/CD4CM = CD8/CD4 central memory T cell; CD8/CD4EM = CD8/CD4 effector memory T cell; CD8/CD4Trm = CD8/CD4 terminal effector memory T cell; MAIT = mucosal-associated invariant T cell; Treg = regulatory T cell
